# Ultra-sensitive detection of prion protein fibrils by flow cytometry in blood from cattle affected with bovine spongiform encephalopathy

**DOI:** 10.1186/1472-6750-5-26

**Published:** 2005-10-04

**Authors:** Lothar Trieschmann, Alexander Navarrete Santos, Katja Kaschig, Sandra Torkler, Elke Maas, Hermann Schätzl, Gerald Böhm

**Affiliations:** 1 ACGT ProGenomics AG, Weinbergweg 22, D-06120 Halle (Saale), Germany; 2 Institute of Virology, Technical University of Munich, Biedersteinerstrasse 29, D-80802 Munich, Germany

## Abstract

**Background:**

The definite diagnosis of prion diseases such as Creutzfeldt-Jakob disease (CJD) in humans or bovine spongiform encephalopathy (BSE) in cattle currently relies on the *post mortem *detection of the pathological form of the prion protein (PrP^Sc^) in brain tissue. Infectivity studies indicate that PrP^Sc ^may also be present in body fluids, even at presymptomatic stages of the disease, albeit at concentrations well below the detection limits of currently available analytical methods.

**Results:**

We developed a highly sensitive method for detecting prion protein aggregates that takes advantage of kinetic differences between seeded and unseeded polymerization of prion protein monomers. Detection of the aggregates was carried out by flow cytometry. In the presence of prion seeds, the association of labelled recombinant PrP monomers in plasma and serum proceeds much more efficiently than in the absence of seeds. In a diagnostic model system, synthetic PrP aggregates were detected down to a concentration of approximately 10^-8 ^nM [0.24 fg/ml]. A specific signal was detected in six out of six available serum samples from BSE-positive cattle.

**Conclusion:**

We have developed a method based on seed-dependent PrP fibril formation that shows promising results in differentiating a small number of BSE-positive serum samples from healthy controls. This method may provide the basis for an *ante mortem *diagnostic test for prion diseases.

## Background

A group of fatal transmissible neurodegenerative diseases, including Creutzfeld-Jakob disease (CJD), bovine spongiform encephalopathy (BSE), chronic wasting disease (CWD) and scrapie, is caused by an unusual infectious agent that has been termed prion [1]. Prions consist of an aberrant isoform (PrP^Sc^) of the normal cellular prion protein (PrP^C^). PrP^C ^is a cell surface glycoprotein expressed in neurons [2] and other cell types [3,4]. The precise physiological function of the cellular prion protein is not known yet. PrP^Sc ^differs from PrP^C ^in its higher content of β-sheet structure [5,6], its partial resistance to protease digestion [7], and its tendency to form large aggregates [8]. PrP^Sc ^propagates by converting the cellular prion protein to the PrP^Sc ^conformation [9]. PrP^Sc ^aggregates accumulate predominantly in the central nervous system (CNS), and definitive diagnosis of prion diseases currently relies on the *post mortem *detection of PrP^Sc ^in CNS tissue by immunohistochemistry, Western blotting, or ELISA [10]. Transmission studies indicate that prions may also be present in blood, potentially allowing for *ante mortem *diagnosis, but the sensitivity of the currently available analytical methods is insufficient for the detection of the extremely low prion titers that can be expected in body fluids [11].

Here, we report the development of a method based on kinetic differences between seeded and unseeded aggregation of prion protein that allows the detection of PrP aggregates in blood down to attomolar concentrations by flow cytometry.

## Results and discussion

### Detection of synthetic prion protein aggregates in serum or plasma

Kinetic differences between seeded and spontaneous polymerization of peptide monomers can be used for the detection of amyloid β-protein aggregates in the cerebrospinal fluid of Alzheimer's disease patients [15]. Here, we extend the principle of seeded polymerization to the detection of prion protein aggregates.

**Figure 1:**
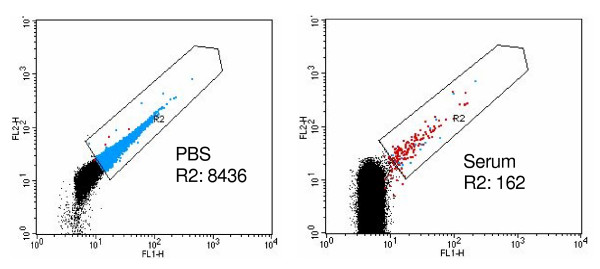
**Inhibition of PrP aggregation in serum**. FITC-labeled recombinant bovine prion protein (concentration 10 nM) was incubated at 37°C for 20 h with continuous shaking, either in 150 μl PBS (left panel) or in the same volume of serum (right panel), followed by flow cytometry. The measurements are depicted in a Fluorescence 1 (FL1-H) vs. Fluorescence 2 (FL2-H) dot-plot. The number of counts in the area containing specific signals (R2) is given in the figures. Aggregate formation in serum is strongly inhibited.

While trying to establish conditions for the labeling of synthetic prion protein aggregates with a fluorescently labeled prion protein probe, we observed that the formation of prion protein aggregates proceeds much less efficiently in serum or plasma (not shown) than in PBS (Fig. 1). This inhibition is probably caused by interactions of the prion protein probe with serum proteins.

**Figure 2:**
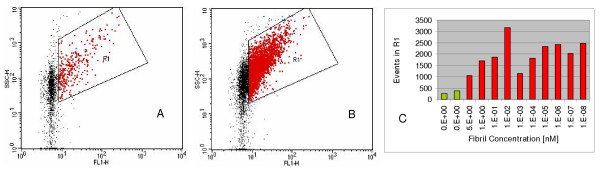
**Seed-dependent PrP aggregate formation in plasma**. FITC-labeled recombinant prion protein (5 nM) was incubated in plasma as described in the methods section for 20 h either in the absence (panel A) or presence (panel B) of 10^-8 ^nM PrP aggregates. Panel C: quantification of measurements shown in A and B, and of measurements (not shown) with different seed concentrations. The measurements are depicted in a Fluorescence 1 (FL1-H) vs. Side-Scatter (SSC) dot-plot. Aggregate formation (signal in region R1) was strongly enhanced by all seed concentrations tested, from 5 nM to 10^-8 ^nM.

Next, we found that the addition of preformed prion protein aggregates to plasma can partially overcome this inhibition (Fig. 2). The preformed aggregates presumably function as seeds that facilitate the formation of new aggregates in the inhibitory environment of plasma. The seeds stimulated the formation of prion protein aggregates at all concentrations tested, from 5 nM [120 ng/ml] to 10^-8 ^nM [0.24 fg/ml] (Fig 2C). The average ratio of event counts in seeded samples to those in samples without seeds was 6.4. The number of events, however, was not proportional to the seed concentration, but remained relatively constant over the whole concentration range. Thus, the seed-dependent formation of prion protein aggregates can be used to detect extremely low amounts (down to the attomolar range) of spiked prion protein aggregates in blood.

### Analysis of serum from clinical-stage, BSE-positive cattle

**Figure 3:**
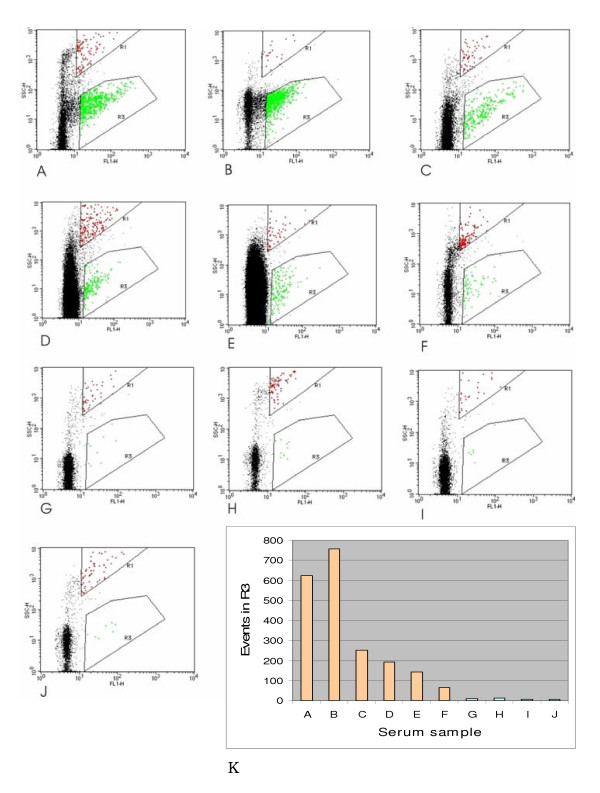
**Analysis of serum from BSE-positive cattle**. FITC-labeled recombinant prion protein (10 nM) was incubated in 150 μl of the serum samples as described in the methods section and analyzed by flow cytometry. The measurements are shown in a Fluorescence 1 (FL1-H) vs. Side-Scatter (SSC) dot-plot. All six BSE-samples (A-F) can be differentiated from the controls (G-J) by a population of events in region R3 (green dots). Panel K: Quantification of measurements shown in panels A-J.

Studies demonstrating the transmission of prion diseases by blood transfusion suggest that prions are present in the blood of afflicted animals and people, even at pre-symptomatic stages of the disease [16-18]. We used the method of seed-dependent fibril formation to analyze serum from six confirmed cases of clinical-stage, BSE-positive cattle and four controls. Based on the spiking experiments described above, our hypothesis was that any PrP^Sc ^aggregates present in serum may act as seeds for the formation of easily detectable amounts of labeled PrP aggregates, whereas in the absence of seeds the formation of PrP aggregates would be inhibited. The serum samples from BSE-positive cattle and controls from healthy cattle were incubated with 10 nM of a FITC-labeled bovine PrP probe at 37°C for 20 h with continuous shaking, followed by analysis in a flow cytometer. All six BSE-samples could be clearly distinguished by a population of events that was absent in the controls (Fig. 3A–J, green dots in region R3; quantification in fig. 3K).

## Conclusion

We have developed a method based on seed-dependent PrP fibril formation that shows promising results in differentiating a small number of BSE-positive serum samples from healthy controls. More samples need to be tested in order to validate its potential as an *ante mortem *diagnostic test for BSE and other prion diseases.

## Methods

### Biological fluids

Serum samples from six confirmed cases of BSE in cattle and four control animals were obtained from BFAV, Insel Riems, Germany. Control plasma was obtained from a blood bank.

### Labeling of prion protein

Recombinant full-length bovine PrP was produced as described previously [12,13]. The purified protein was labeled with a FITC-labeling kit (Roche) according to the manufacturer's instructions.

### Preparation of fibrils from recombinant prion protein

25 μM of unlabeled bovine prion protein in PBS containing 0.2 % SDS was incubated for 10 min at room temperature, followed by a twentyfold dilution with PBS. For fibril formation, the diluted reaction mixture was incubated for 48 h at room temperature [14].

### PrP fibril formation in serum or plasma

Recombinant FITC-labeled bovine prion protein was incubated in 150 μl serum or plasma at a concentration of 5 or 10 nM for 5–10 min. at 20°C, shaking at 550 rpm in an Eppendorf thermomixer, followed by an increase of the temperature to 37°C h at constant shaking speed. The incubation was continued for 20 h. Samples were then analyzed by flow cytometry.

### Flow cytometry

Analysis of the samples was carried out on a FACSVantage flow cytometer (BD Biosciences) at room temperature, measurement time was 30 sec per sample.

## Authors' contributions

LT participated in the design of the study, carried out the measurements and drafted the manuscript. ANS participated in the analysis of the data. EM prepared the recombinant protein. KK and ST were also involved in protein expression and purification. HS participated in the design and coordination of the study. GB conceived of the study and helped to draft the manuscript. All authors read and approved the final manuscript.

## References

[B1] Prusiner SB (1982). Novel proteinaceous infectious particles cause scrapie. Science.

[B2] Kretzschmar HA, Prusiner SB, Stowring LE, DeArmond SJ (1986). Scrapie prion proteins are synthesized in neurons. Am J Pathol.

[B3] Cashman NR, Loertscher R, Nalbantoglu J, Shaw I, Kascsak RJ, Bolton DC, Bendheim PE (1990). Cellular isoform of the scrapie agent protein participates in lymphocyte activation. Cell.

[B4] Manson J, West JD, Thomson V, McBride P, Kaufman MH, Hope J (1992). The prion protein gene: a role in mouse embryogenesis?. Development.

[B5] Pan KM, Baldwin M, Nguyen J, Gasset M, Serban A, Groth D, Mehlhorn I, Huang Z, Fletterick RJ, Cohen FE, Prusiner SB (1993). Conversion of alpha-helices into beta-sheets features in the formation of the scrapie prion proteins. Proc Natl Acad Sci U S A.

[B6] Caughey BW, Dong A, Bhat KS, Ernst D, Hayes SF, Caughey WS (1991). Secondary structure analysis of the scrapie-associated protein PrP 27–30 in water by infrared spectroscopy. Biochemistry.

[B7] Prusiner SB, Groth DF, Bolton DC, Kent SB, Hood LE (1984). Purification and structural studies of a major scrapie prion protein. Cell.

[B8] Prusiner SB, McKinley MP, Bowman KA, Bolton DC, Bendheim PE, Groth DF, Glenner GG (1983). Scrapie prions aggregate to form amyloid-like birefringent rods. Cell.

[B9] Prusiner SB (1998). Prions. Proc Natl Acad Sci U S A.

[B10] Kretzschmar HA, Ironside JW, DeArmond SJ, Tateishi J (1996). Diagnostic criteria for sporadic Creutzfeldt-Jakob disease. Arch Neurol.

[B11] Brown P, Cervenakova L, Diringer H (2001). Blood infectivity and the prospects for a diagnostic screening test in Creutzfeldt-Jakob disease. J Lab Clin Med.

[B12] Proske D, Gilch S, Wopfner F, Schätzl HM, Winnacker EL, Famulok M (2002). ion-protein-specific aptamer reduces PrP^Sc ^formation. Chembiochem.

[B13] Gilch S, Wopfner F, Renner-Muller I, Kremmer E, Bauer C, Wolf E, Brem G, Groschup MH, Schätzl HM (2003). Polyclonal anti-PrP auto-antibodies induced with dimeric PrP interfere efficiently with PrP^Sc ^propagation in prion-infected cells. J Biol Chem.

[B14] Post K, Pitschke M, Schafer O, Wille H, Appel TR, Kirsch D, Mehlhorn I, Serban H, Prusiner SB, Riesner D (1998). Rapid acquisition of beta-sheet structure in the prion protein prior to multimer formation. Biol Chem.

[B15] Pitschke M, Prior R, Haupt M, Riesner D (1998). Detection of single amyloid beta-protein aggregates in the cerebrospinal fluid of Alzheimer's patients by fluorescence correlation spectroscopy. Nat Med.

[B16] Llewelyn CA, Hewitt PE, Knight RS, Amar K, Cousens S, Mackenzie J, Will RG (2004). Possible transmission of variant Creutzfeldt-Jakob disease by blood transfusion. Lancet.

[B17] Peden AH, Head MW, Ritchie DL, Bell JE, Ironside JW (2004). Preclinical vCJD after blood transfusion in a PRNP codon 129 heterozygous patient. Lancet.

[B18] Hunter N, Foster J, Chong A, McCutcheon S, Parnham D, Eaton S, MacKenzie C, Houston F (2002). Transmission of prion diseases by blood transfusion. J Gen Virol.

